# Value and Efficacy of Transcranial Direct Current Stimulation in the Cognitive Rehabilitation: A Critical Review Since 2000

**DOI:** 10.3389/fnins.2016.00157

**Published:** 2016-04-18

**Authors:** Davide Cappon, Marjan Jahanshahi, Patrizia Bisiacchi

**Affiliations:** ^1^Department of General Psychology, Center for Cognitive Neuroscience, University of PadovaPadua, Italy; ^2^Sobell Department of Motor Neuroscience and Movement Disorders, Institute of Neurology, University College LondonLondon, UK

**Keywords:** transcranial direct current stimulation (tDCS), cognitive rehabilitation, neurocognitive disorders, unilateral neglect, Aphasia, Parkinson's disease (PD), Alzheimer's disease (AD)

## Abstract

Non-invasive brain stimulation techniques, including transcranial direct current stimulation (t-DCS) have been used in the rehabilitation of cognitive function in a spectrum of neurological disorders. The present review outlines methodological communalities and differences of t-DCS procedures in neurocognitive rehabilitation. We consider the efficacy of tDCS for the management of specific cognitive deficits in four main neurological disorders by providing a critical analysis of recent studies that have used t-DCS to improve cognition in patients with Parkinson's Disease, Alzheimer's Disease, Hemi-spatial Neglect, and Aphasia. The evidence from this innovative approach to cognitive rehabilitation suggests that tDCS can influence cognition. However, the results show a high variability between studies both in terms of the methodological approach adopted and the cognitive functions targeted. The review also focuses both on methodological issues such as technical aspects of the stimulation (electrode position and dimension; current intensity; duration of protocol) and on the inclusion of appropriate assessment tools for cognition. A further aspect considered is the optimal timing for administration of tDCS: before, during or after cognitive rehabilitation. We conclude that more studies using common methodology are needed to gain a better understanding of the efficacy of tDCS as a new tool for rehabilitation of cognitive disorders in a range of neurological disorders.

## Introduction

The fifth edition of the Diagnostic and Statistical Manual of Mental Disorders (DSM-5) presented a structure for the diagnosis of neurocognitive disorders. It differentiated “mild” and “major” neurocognitive disorders which may be due to diverse etiologies (Sachdev et al., 2014). Neurocognitive disorders (NCD) are described by decline from a premorbidly reached level of cognitive functioning. The NCD category includes distinct clinical characteristics in which the primary clinical deficit is acquired and is in cognitive function. The prevalence of NCD increases exponentially with age and at the present moment there are no effective pharmacological treatments for these cognitive deficits. Thus, in the context of rapid population aging worldwide, it becomes important to find new strategies to deal with NCD. Specifically, Parkinson's Disease, Alzheimer's Disease, Vascular Disease are particularly debilitating conditions with cognitive sequelae which have increased in prevalence over the years and are a burden for society.

In the last decades non-invasive brain stimulation (NIBS) techniques have rapidly become an important approach as potential therapeutic tools to improve the outcome of cognitive rehabilitation in patients affected by stroke, neurodegenerative disorders, or psychiatric diseases (Rossini et al., 2015). The two most commonly used techniques for non-invasive brain stimulation (NIBS) are transcranial magnetic stimulation (TMS) (including single pulse TMS, repetitive(rTMS) and theta burst TMS) and transcranial electrical stimulation (tES) (including transcranial direct current stimulation (tDCS), high-definition tDCS, transcranial alternating current stimulation (tACS), transcranial random noise stimulation (tRNS; Peterchev et al., 2012). NIBS apply different electromagnetic principles to non-invasively influence neural activity: TMS involves neurostimulation and neuromodulation of neural tissue, including cerebral cortex, spinal roots, and cranial and peripheral nerves, whereas tES is a purely neuromodulatory intervention (Rothwell, 1997). In other words, tDCS using weak current, unlike TMS is not able to discharge resting axons to produce action potentials, although it can be used to modulate cortical excitability. In tDCS surface electrodes (anode and cathode) inject low amplitude direct current (0.5–2 mA) through the scalp and brain. In early studies tDCS was combined with TMS to investigate modification of primary motor cortex cortical excitability by recording motor evoked potentials (MEPs) (Priori et al., 1998; Nitsche and Paulus, 2000). The mechanisms are not yet clear but presumably the current induces changes in the resting membrane potential of neurons. These changes appear to be polarity specific with anodal depolarization and cathodal hyperpolarization of resting membrane potential (Nitsche and Paulus, 2000; Nitsche et al., 2003). Some studies have been performed in order to understand the physiological mechanisms and it seems that neuroplastic after-effects are N-methyl-D-aspartate (NMDA) receptor dependent (Liebetanz et al., 2002; Nitsche et al., 2004). In fact, it has been shown that the effects can be modified, prolonged or even reversed by drugs acting on the central nervous system (Stagg and Nitsche, 2011). It is noteworthy that NMDA receptors have been reported to have a critical role in synaptic plasticity and long term potentiation (LTP) affecting learning and memory. However, these studies are in the motor domain and it is still not clear to what extent these findings are transferable to other areas of the brain.

Nonetheless, during the last decade a growing body of experimental work have extensively explored the effects of tDCS on brain areas other than the primary motor cortex with encouraging results. These studies have demonstrated significant effects of tDCS on cognitive processes as assessed by a variety of cognitive tasks not only in healthy participants but also in clinical populations. As a consequence, there has been growing interest to use tDCS as a safe and relatively low-cost technique for neurological and neuropsychological rehabilitation as demonstrated by recent reviews of this topic for various cognitive deficits (Fasotti and van Kessel, 2013; Elder and Taylor, 2014; Flöel, 2014; de Aguiar et al., 2015).

The present paper intends to review recent evidence of tDCS for neurocognitive rehabilitation. Our first aim is to discuss the key issues that have emerged from the studies that have demonstrated potential therapeutic applications of t-DCS in neurocognitive disorders. Four clinical conditions will be considered namely Parkinson's Disease, Alzheimer's Disease, Unilateral Hemispatial Neglect and Aphasia. The second aim is give the reader an illustration of the methodological communalities and differences of the studies published so far. Finally, we propose a framework of factors that should be taken into account for an increased understanding of the functional role of tDCS in improving symptoms in patients suffering from neurocognitive disorders.

## Methods

**Figure 1:**
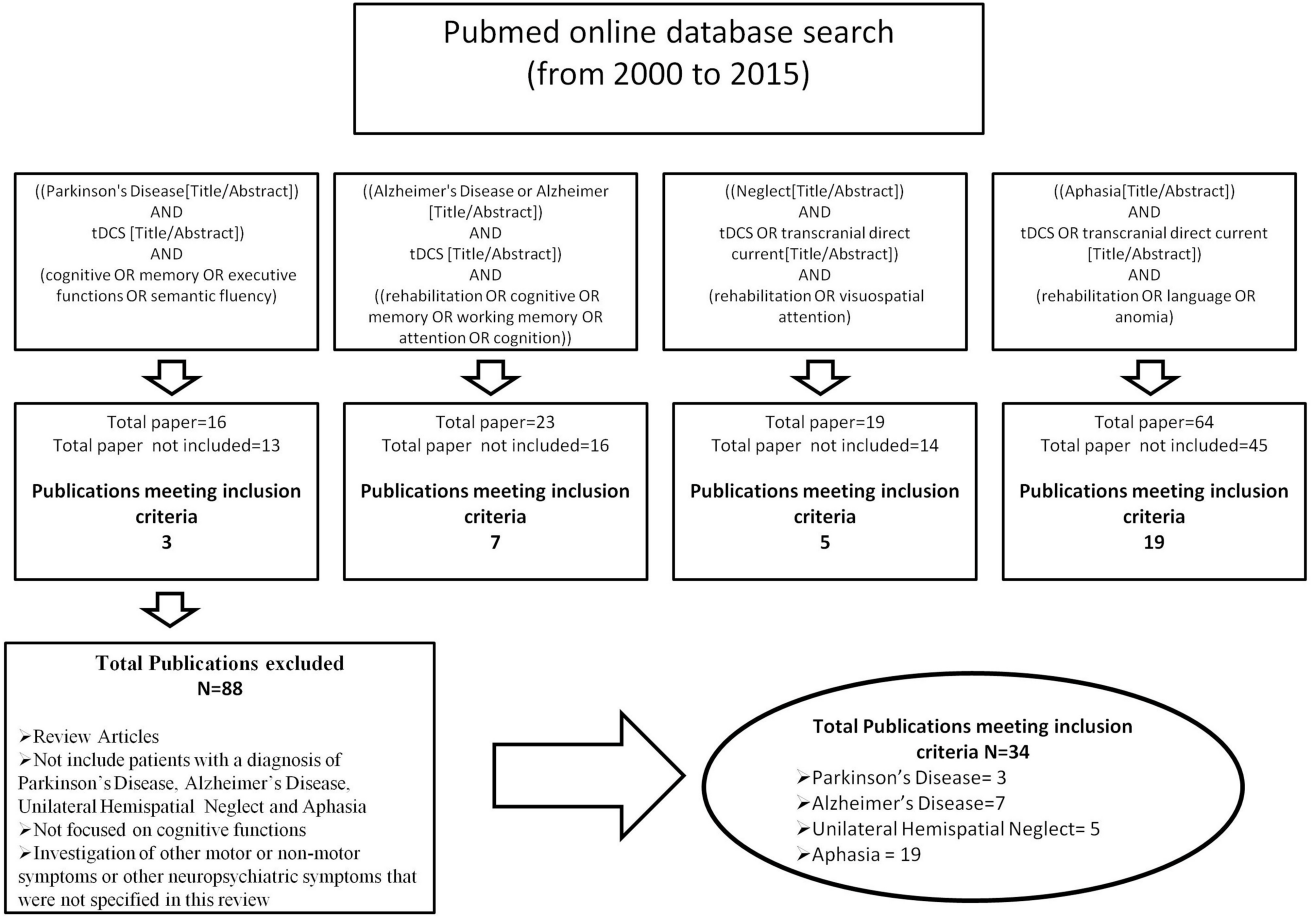
**Database key words search strategy**.

Searches were conducted using the online database Pubmed and manual searches of references in relevant papers. The review period was from 2000 to 2015. Articles were identified by carrying out a comprehensive review of published research papers that have used tDCS to improve cognition in patients with Parkinson's Disease, Alzheimer's Disease, Unilateral Hemispatial Neglect and Aphasia. Search terms were ((Parkinson's Disease[Title/Abstract]) AND tDCS [Title/Abstract]) AND (cognitive OR memory OR executive functions OR semantic fluency); ((Alzheimer's Disease or Alzheimer [Title/Abstract]) AND tDCS [Title/Abstract]) AND ((rehabilitation OR cognitive OR memory OR working memory OR attention OR cognition)); ((Neglect[Title/Abstract]) AND tDCS OR transcranial direct current[Title/Abstract]) AND (rehabilitation OR visuospatial attention); ((Aphasia[Title/Abstract]) AND tDCS OR transcranial direct current [Title/Abstract]) AND (rehabilitation OR language OR anomia). The initial search identified 122 titles and abstracts. The abstracts and full paper were reviewed to eliminate articles according to the following exclusion criteria: (1) review articles (2) papers that did not include patients with a diagnosis of Parkinson's Disease, Alzheimer's Disease, Hemi-spatial Neglect or Aphasia (3) studies that did not focus on cognitive abilities (4) the investigation of other non-motor symptoms or other neuropsychiatric symptoms that were not specified in this review. In total 34 articles met our inclusion criteria (Figure 1).

## Application of t-DCS for cognitive rehabilitation

**Figure 2:**
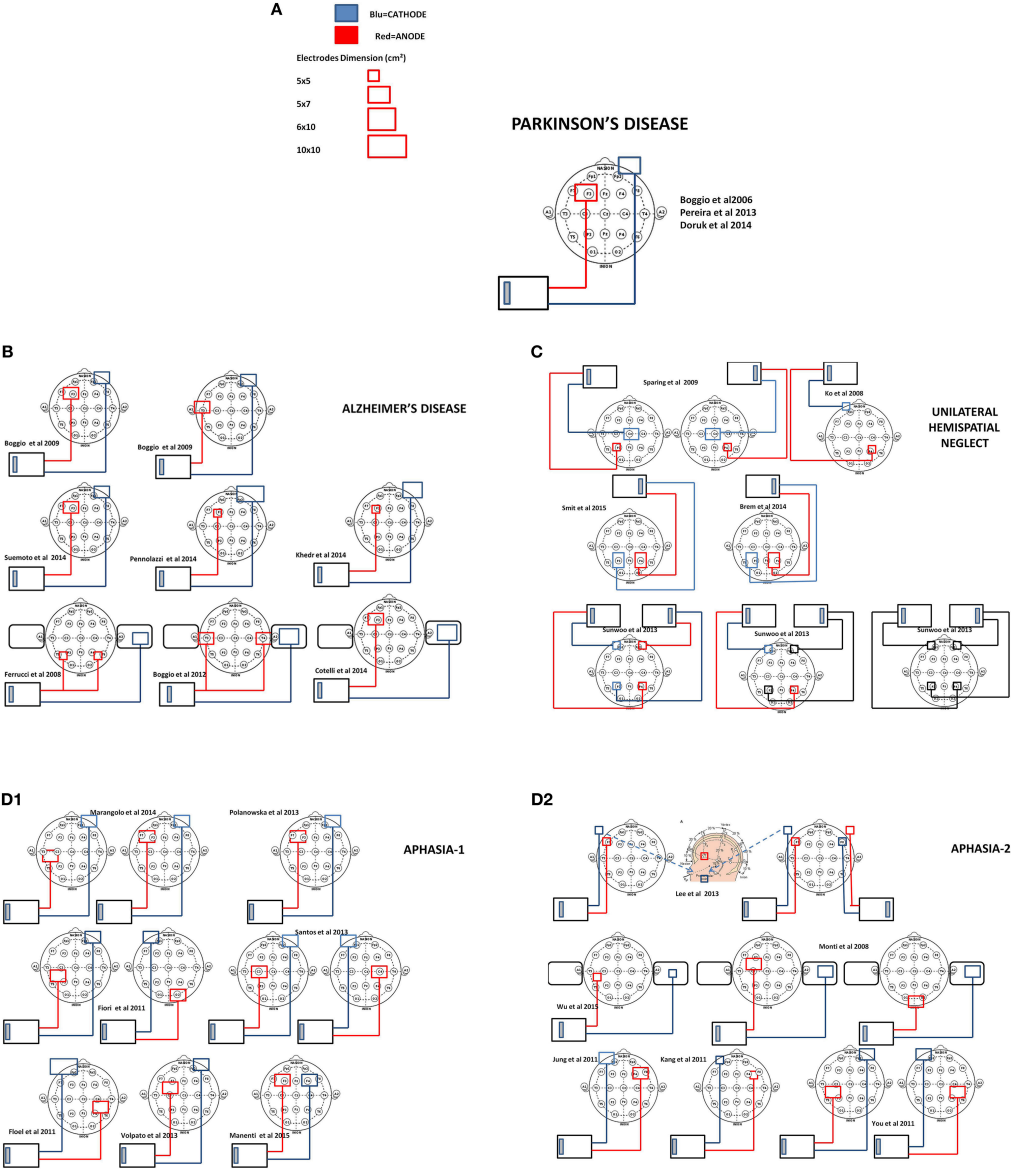
**Scale representation of tDCS electrode montage of the reviewed studies with reference to the EEG international 10–20 system**. In **(A)** legend of electrodes size and polarity and electrode montage in Parkinson's disease studies (Boggio et al., 2006; Pereira et al., 2013; Doruk et al., 2014), **(B)** Alzheimer's disease (Boggio et al., 2008, 2012; Ferrucci et al., 2008; Cotelli et al., 2014; Khedr et al., 2014; Penolazzi et al., 2014; Suemoto et al., 2014), **(C)** unilateral Neglect (Ko et al., 2008; Sparing et al., 2009; Sunwoo et al., 2013; Brem et al., 2014; Smit et al., 2015), and **(D1,D2)** Aphasia (Monti et al., 2008; Flöel et al., 2011; Fiori et al., 2011; Jung et al., 2011; Kang et al., 2011; You et al., 2011; Lee et al., 2013; Polanowska et al., 2013; Santos et al., 2013; Volpato et al., 2013; Marangolo et al., 2014; Manenti et al., 2015; Wu et al., 2015).

In this section we will review evidence on the use of tDCS for cognitive rehabilitation in patients with Parkinson's Disease, Alzheimer's Disease, Hemispatial Neglect or Aphasia. For each disorder we start with a concise description of the main features of cognitive deficit, followed by a detailed review of the studies. The methodological details of parameters of stimulation used in these studies are presented in Table 1. Patient characteristics, experimental design, cognitive domains targeted, tasks used as outcome measures and main results are summarized in Table 2. In Figures 2A–D is a visual representation of the electrode montage which could be useful to compare the studies.

**Table 1:** **Parameters of stimulation in studies of t-DCS for cognitive rehabilitation in Parkinson's disease, Alzheimer's disease, unilateral spatial neglect or aphasia**.

**Author and year**	**Electrodes position**		**Electrodes dimension (cm**^**2**^**)**		**Current**			**Duration (min)**	**Sessions**	**Time of stimulation**
	**“Active”**	**“Reference”**	**“Active”**	**“Reference”**	**Intensity (mA)**	**Density (mA/cm**^**2**^**)**				
						**“Active”**	**“Reference”**			
**PARKINSON's DISEASE**										
Boggio et al. (2006)	(1)Anode L-DLPFC (2)AnodeM1	Cathode C.F. Cathode C.F.	35	35	1 2	0.029 0.057	0.029 0.057	20	1	Online partially during WM task
Pereira et al. (2013)	(1)Anode L-DLPFC (2)Anode L-TPC	Cathode C.F. Cathode C.F.	35	35	2	0.057	0.057	20	1	Rest
Doruk et al. (2014)	(1)Anode L-DLPFC (2)Anode R-DLPFC	Cathode C.F. Cathode C.F.	35	35	2	0.057	0.057	20	10 (2 weeks)	Rest
**ALZHEIMER's DISEASE**										
Ferrucci et al. (2008)	(1)Anode L-TPC R-TPC bilaterally (2)Cathode L-TPC R-TPC bilaterally	R-Deltoide	25 25	25	1.5	0.060	0.060	15	1	Rest
Boggio et al. (2008)	(1)Anode L-DLPFC (2)Anode L-TC (T7)	Cathode C.F. Cathode C.F.	35	35	2	0.057	0.057	30	1	Online
Boggio et al. (2012)	Anode L-TPC R-TPC bilaterally (T3-T4)	R-Deltoide	35 35	64	2	0.057 0.057	0.031	30	5	Rest
Cotelli et al. (2014)	Anode L-DLPFC	R-Deltoide	25	60	2	0.080	0.033	25	10 (2 weeks)	Online
Khedr et al. (2014)	(1)Anode L-DLPFC (2)Cathode L-DLPFC	Cathode C.F. Anode C.F.	24	100	2	0.083	0.010	25	10	Rest
Suemoto et al. (2014)	Anode L-DLPFC	Cathode C.F.	35	35	2	0.057	0.057	20	6 (2weeks)	Rest
Penolazzi et al. (2014)	Anode L-DLPFC	Cathode C.F.	35	100	2	0.057	0.010	20	10 (2 weeks)	Rest
**UNILATERAL HEMISPATIAL NEGLECT**										
Ko et al. (2008)	Anode R-PPC (P4)	Cathode C.F.	25	25	2	0.080	0.080	20	1	Rest
Sparing et al. (2009)	(1)Anode R-PPC (P4) (2) Cathode L- PPC (P3)	Cathode Cz	25	35	1	0.040	0.029	10	1	Rest
Sunwoo et al. (2013)	(1)Dual-mode, Anode R- PPC Cathode L- PPC; (2)Single-mode, R Anode R- PPC	(1)Cathode C.F.; Anode C.F. (2)Cathode C.F.	25	25	1	0.040	0.040	20	1	Rest
Brem et al. (2014)	Anode R-PPC	Cathode L-PPC	35	35	1	0.029	0.029	20	5	Online
Smit et al. (2015)	Anode R-PPC	Cathode L-PPC	n.a	n.a	2	n.a	n.a	20	5	Rest
**APHASIA**										
Monti et al. (2008)	(1)Anode Broca's area (between T3-Fz and F7-Cz) Cathode Broca's area (between T3-Fz and F7-Cz) (2) Cathode occipital areas (2 cm over the inion)	Cathode R Deltoide Anode R Deltoide Anode R Deltoide	35	35	2	0.057	0.057	10	1	Rest
Baker et al. (2010)	Anode LFC Individually determined (fMRI task)	Cathode R-Deltoide	25	25	1	0.040	0.040	20	5	Online computerized anomia training
Fiori et al. (2011)	Anode L Wernicke's area	Cathode C.F.	35	35	1	0.029	0.029	20	5	Online picture-naming task
Flöel et al. (2011)	(1)Anode R-TPC (Talairach) (2)Cathode R-TPC (Talairach)	(1)Cathode C.F. (2)Anode C.F.	35	100	1	0.029	0.010	20		Online during the first 20 min anomia training
Fridriksson et al. (2011)	Anode LPC Individually determined	Cathode C.F.	25	25	1	0.040	0.040	20	5	Online computerized anomia treatment
Jung et al. (2011)	Cathode R BA 45(between T4-Fz and F8-Cz)	Anode C.F.	35	35	1	0.029	0.029	20	10	Online speech therapy
Kang et al. (2011)	Cathode R- Broca's area	Anode C.F.	25	25	2	0.080	0.080	20	5	Online word-retrieval training
Vines et al. (2011)	Anode R IFG, (2.5 cm posterior to F8)	Cathode C.F.	16	30	1.2	0.075	0.040	20	3	Online Melodic intonation therapy
You et al. (2011)	(1)Anode L sTG (CP5) (2)Cathode R sTG (CP6)	(1)Cathode C.F. (2)Anode C.F.	35	35	2	0.057	0.057	30	10 (2 weeks)	Online speech and language therapy
Lee et al. (2013)	(1)single, Anode L IFG (F7) (2) dual, Anode L IFG (F8) Cathode R IFG	(1)Cathode L buccinator muscle (2)Cathode L buccinator muscle Anode R buccinator muscle	25	25	2	0.080	0.080	30	1	Online speech therapy during the last 15 min
Polanowska et al. (2013)	Anode L-Broca's area (T3-Fz and F7-Cz)	Cathode C.F.	35	35	1	0.029	0.029	10	15	Rest (followed by 45 min language training)
Rosso et al. (2014)	Cathode R Broca's area (Individually determined,neuronavigator)	Anode C.F.	35	35	1	0.029	0.029	15	1	Rest
Santos et al. (2013)	Cathode M1of unaffected side (C3/C4)	Anode C.F.	35	35	2	0.057	0.057	20	10	Rest
Volpato et al. (2013)	Anode L-Broca's Area (between T3-Fz and F7-Cz)	Cathode C.F.	35	35	2	0.057	0.057	20	10 (2 weeks)	Rest
Marangolo et al. (2014)	(1)Anode L Wernicke's area (2)Anode L Broca's area	Cathode C.F.	35	35	1	0.029	0.029	20	5	Offline training for action naming
Vestito et al. (2014)	LF perilesional site (between T3-Fz and F7-Cz)	Cathode C.F.	25	25	1.5	0.060	0.060	20	10 (2weeks)	Online naming training
Manenti et al. (2015)	Anode L-DLPFC (F3)	Cathode R-DLPFC (F4)	35	35	2	0.057	0.057	25	20	Online verb anomia training
Shah-Basak et al. (2015)	Individualized on the individual response (1)Anode L-IFG(F3) (2)Cathode L-IFG (F3) (3)Anode R-IFG (F4) (4)Cathode R-IFG (F4)	Controlateral Mastoide	25	25	2	0.080	0.080	20	10 (2weeks)	Online picture-naming task
Wu et al. (2015)	Anode L Wernicke's area (between T3-P3 and C3-T5)	Cathode unaffected shoulder	25	25	1.2	0.048	0.048	20	20	Rest

*BA, Broadmann area; C.F., controlateral forehead; L, left; R, right; LF, left-frontal; DLPFC, dorso lateral prefrontal cortex; IFG, inferior frontal gyrus; mA, milliAmpere; min, minutes; M1, primary motor cotex; TC, temporal cortex; TPC, temporo-parietal cortex; PPC, posterior parietal cortex; sTG, superior temporal gyrus*.

**Table 2:** **Patient characteristics, experimental design, cognitive domains, tasks used as outcome measures and main results of studies which used tDCS for cognitive rehabilitation in Parkinson's disease, Alzheimer' disease, unilateral neglect, or aphasia**.

**Author and year**	**Sample**	**Experimental design**	**Target cognitive domain**	**Neuropsychological measures**	**Main results**
**PARKINSON's DISEASE**					
Boggio et al. (2006)	Idiopathic Parkinson *N* = 18	Randomized controlled cross over	Working Memory	Computerized 3 n-back task	A-tDCS (2mA) of left DLPFC improved accuracy as compared with the other conditions
Pereira et al. (2013)	Idiopathic Parkinson *N* = 16	Randomized controlled cross over	Executive Functions	Computerized verbal fluency task (phonemic fluency, semantic fluency)	A-tDCS L-DLPFC improved performance on the phonemic fluency task as compared L-TPC A-tDCS
Doruk et al. (2014)	Idiopathic Parkinson *N* = 18	Randomized controlled between subject	Abstract Reasoning Executive Functions Selective Attention Visuo-spatial abilities Working Memory	TMT A-B, WCST, DIGSP-BW- FW, HPVOT,CPM, Stroop Test	Both left and right DLPFC A-tDCS groups improved at the 1-month follow-up in TMT-B as compared with sham; no changes in WSCT, PCL, WM, CPM, HVOT,STROOP, and Digit Span
**ALZHEIMER's DISEASE**					
Ferrucci et al. (2008)	AD *N* = 10 (criteria MMSE≥20)	Randomized controlled cross over	Episodic Memory Attention	word recognition task visual attention task	Improvement of accuracy of word recognition memory after A-tDCS; no changes in visual attention
Boggio et al. (2008)	AD *N* = 10 (criteria 12<MMSE<25)	Randomized controlled cross over	Executive Functions Selective Attention Working Memory	visual recognition, DIGSP-BW- FW, Stroop	Improvement of visual recognition memory after both temporal and prefrontal A-tDCS;no changes in stroop and digit span
Boggio et al. (2012)	AD *N* = 15 (MMSE>15)	Randomized controlled cross over	Executive Functions Selective Attention Working Memory Global Functioning	Computerized recognition memory task, visual attention task, ADAS-cog, MMSE	Improvement of visual recognition memory after A-tDCS persist for 4 weeks; no changes in other measures
Cotelli et al. (2014)	AD *N* = 36 (Mild to moderate AD)	Randomized controlled between subject	Attention Episodic Memory Executive Functions Functional status Language Praxia Semantic Memory	Computerized Face-name association task, MMSE, ADL, IADL, Picture naming task, BADA, RBMT, RAVLT, ROCFC, TMT A-B	Both sham and real tDCS led to improvement in FNAT performance; persist 12 weeks only for the placebo group. no changes in other measures
Khedr et al. (2014)	AD *N* = 34 (criteria 12<MMSE<23)	Randomized controlled between subject	Global Functioning Intelligence	MMSE,WAIS-III	both A-tDCS and C-tDCS improved MMSE in contrast to sham; only C-tDCS improved performance in the subscales of WAIS-III
Suemoto et al. (2014)	AD *N* = 40 (criteria 10<MMSE<20)	Randomized controlled cross over	Global Functioning	MMSE,ADAS-COG	No effects of repetitive A-tDCS L- DLPFC on cognitive measure tested
Penolazzi et al. (2014)	AD *N* = 1 (MMSE = 23)	Single-case controlled cross over	Episodic Memory Executive Functions Working Memory Selective Attention Praxia Visuo-spatial abilities	Computerized word and visual recognition, verbal fluency, CPT, ENB-2	A-tDCS+CT condition had few effects on the cognitive measures; A-tDCS+CT induced a stability of the patient's global cognitive functioning lasting 3 months as compare to sham+CT condition
**UNILATERAL HEMISPATIAL NEGLECT**					
Ko et al. (2008)	Subacute stroke Neglect *N* = 15	Randomized controlled Cross-over	Neglect Visuo-spatial search Attention	Line bisection, letter and figure cancelation	A-tDCS compare to sham improved both neglect tests performance.
Sparing et al. (2009)	Subacute and chronic stroke Neglect *N* = 10	Randomized controlled Cross-over	Neglect Visuo-spatial search Attention	Computerized line bisection and visual detection tasks	C-tDCS over the unlesioned hemisphere and A-tDCS over lesioned hemisphere reduced symptoms of visuospatial neglect
Sunwoo et al. (2013)	Chronic stroke *N* = 10	Randomized controlled cross over	Neglect Visuo-spatial search Attention	Line Bisection test, Star cancelation test	Both dual- and the single-mode tDCS improved performance in the line bisection test as compare to sham. No changes in the star cancelation test
Brem et al. (2014)	Subacute stroke Neglect *N* = 1	Single-case controlled double-blind	Neglect Visuo-spatial search Attention	TAP,NET,ADL	Biparietal tDCS stimulation, improved covert attention allocation toward left-sided invalid stimuli, line bisection and copying as compared to sham stimulation
Smit et al. (2015)	Chronic stroke *N* = 5	Double-blind randomized controlled cross-over	Neglect Visuo-spatial search Attention	BIT	No A-tDCS effects were observed for the BIT subtests
**APHASIA**					
Monti et al. (2008)	Chronic stroke Non-fluent aphasia *N* = 8 (Broca's *N* = 4; Global *N* = 4)	Randomized controlled Cross-over	Language (naming abilities)	Computerized overt picture naming task	C-tDCS improved accuracy in picture naming as compare to sham and A-tDCS
Baker et al. (2010)	Chronic stroke *N* = 10 (Anomic aphasia *N* = 6; Broca's aphasia *N* = 4)	Randomized controlled Cross-over	Language (naming abilities)	Computerized picture-word matching task	A-tDCS improved naming accuracy as compared to sham; improvement persist after 1 week
Fiori et al. (2011)	Chronic stroke *N* = 3 Non-fluent aphasia	Double-blind randomized controlled cross-over	Language (naming abilities)	Object naming	A-tDCS improved naming accuracy and RTs as compared to sham; improvement persist after 3 weeks in two patients
Flöel et al. (2011)	Chronic stroke Aphasia (type n.a.) *N* = 12	Randomized controlled Cross-over	Language (naming abilities)	Computerized naming task	Both A-tDCS and C-tDCS improved naming accuracy; effects of A-tDCS persist after 2 weeks
Fridriksson et al. (2011)	Chronic stroke Fluent aphasia *N* = 8	Randomized controlled Cross-over	Language (naming abilities)	Verbal word-picture matching task	A-tDCS improved naming RTs as compared to sham; improvement persist after 3 weeks
Jung et al. (2011)	Acute, subacute, chronic stroke Aphasia *N* = 37	Pretest-Posttest Design (no sham control group)	Language	Aphasia quotient and Korean Western Aphasia Battery	C-tDCS improved aphasia symptoms
Kang et al. (2011)	Chronic stroke Aphasia *N* = 10 Global (*n* = 3), Broca's (*n* = 4), anomic (=2), tanscortical motor (*n* = 1)	Double-Blind Randomized controlled Cross-over	LF (naming abilities)	Naming, picture-word Matching task	C-tDCS improved naming accuracy as compared to sham
Vines et al. (2011)	Chronic stroke Moderate to severe Non-fluent aphasia *N* = 6	Randomized controlled Cross-over	Language (naming abilities) (verbal fluency)	Verbal fluency tasks, picture description and picture naming.	A-tDCS improved speech fluency as compared to sham
You et al. (2011)	Subacute stroke Global Aphasia *N* = 21	Randomized controlled between subject	Language (Auditory Verbal Comprehension)	Auditory Verbal Comprehension	C-tDCS improved auditory verbal comprehension as compared to A-tDCS and sham
Lee et al. (2013)	Chronic stroke Aphasia *N* = 11 (Broca's *N* = 4; Anomic *N* = 5; Transcortical Motor *N* = 2)	Randomized controlled Cross-over	Language (naming abilities)	Picture naming test and picture description	Both single and dual tDCS condition improved naming accuracy and RTs as compared to sham
Polanowska et al. (2013)	Subacute stroke Aphasia (moderate to severe) *N* = 37	Randomized, double-blind, controlled	Language	Boston Diagnostic Aphasia Examination	No differences between A-tDCS and sham group (both improved)
Rosso et al. (2014)	Chronic stroke two groups with (*N* = 11) or without (*N* = 14) infarction in the L-Broca's area. Non-fluent aphasia	Randomized controlled cross-over	Language (naming abilities)	Computerized picture-naming task	C-tDCS improved picture naming accuracy in the group with lesion in the L- Broca's area as compared to the other group
Santos et al. (2013)	Chronic stroke Aphasia *N* = 19 (Broca's *N* = 8;Anomic *N* = 7; Mixed *N* = 4)	Pretest-Posttest Design (no sham control group)	Language (oral comprehension, writing, naming and verbal fluency)	Oral language comprehension, copying, dictation, reading, writing, naming and verbal fluency	A-tDCS improved comprehension, naming and verbal fluency for animals name; no changes in other outcomes
Volpato et al. (2013)	Chronic stroke *N* = 8 aphasia (Wernike's *N* = 2; Broca's *N* = 1;Anomic *N* = 2; Transcortical sensory = 1; Transcortical Motor *N* = 1; Conduction *N* = 1)	Randomized controlled Cross-over	Language (naming abilities)	Computerized picture naming task	No differences between A-tDCS and sham for object and action naming task
Marangolo et al. (2014)	Chronic stroke *N* = 7 Non-fluent aphasia	Randomized controlled Cross-over	Language (naming abilities)	Computerized action naming task	A-tDCS on Broca's area improved naming accuracy as compared with sham; the effects persist at follow-up 1 week and 4weeks
Vestito et al. (2014)	Chronic stroke Aphasia *N* = 3	controlled Cross-over	Language (naming abilities)	Computerized picture naming task	A-tDCS improved naming accuracy as compared to sham; improvement persist after 16 weeks
Manenti et al. (2015)	Chronic stroke non-fluent aphasia *N* = 1	Pre test-Post test Design (no control group)	Language (naming abilities)	Word verb naming	Bi-hemispheric DLPFC tDCS improve verb-naming performances
Shah-Basak et al. (2015)	Chronic stroke non-fluent aphasia (mild to severe) *N* = 12	Randomized controlled Cross-over	Language (naming abilities)	Computerized picture naming task	C-tDCS improved naming as compared to sham
Wu et al. (2015)	Subacute stroke *N* = 12	Randomized controlled Cross-over	Language (naming abilities) (comprehension)	Computerized picture naming auditory word-picture identification	A-tDSC improved picture naming and auditory identification as compared with sham

*Randomized Controlled Cross Over, over time, each participant receives an intervention in a random sequence*.

*Randomized controlled between subject, the various experimental treatments are given to different groups of subjects*.

*tDCS, transcranial direct current stimulation; A-tDCS, anodal electrode tDCS; C-tDCS, cathodal electrode tDCS; sham, placebo tDCS; ADAS-cog, Alzheimer's Disease Assessment Scale-cognitive subscale; ADL, activities of daily living; BADA, Batteria per l'Analisi dei Deficit Afasici; BIT, behavioral inattention test; CPM, colored progressive matrices; DIGSP-BW- FW, digit span backwards-forwards; ENB-2, Esame Neuropsicologico Breve-2; FNAT, face-naming association task; HPVOT, Hooper Visual Organization Test; IADL, Indice di dipendenza nelle attività strumentali della vita quotidiana; MMSE, Mini Mental State Examination; NET, Neglect-Test; RAVLT, Rey auditory verbal learning test; RBMT, River mead behavioral memory test; ROCFC, Rey osterrieth Complex figure copy; TAP, Test for Attentional Performance; TMT A-B, Trail making test A-B;WCST, Wisconsin card sorting test; WAIS-III, Wechsler Adult Intelligence Scale-Third edition*.

### Parkinson's disease (PD)

PD is a chronic and progressive neurodegenerative disorder. PD affects one out of 100 people who are aged older than 60 years in industrialized countries. PD primarily affects dopamine producing neurons in an area of the brain called the substantia nigra pars compacta. The loss of these specific neurons causes motor symptoms characterized by resting tremor, rigidity, bradykinesia and postural instability. These symptoms are the basis for a diagnosis of PD. Mild Neurocognitive disorders (mNCDs) are also common in PD even in the earliest stages of the disease and significantly impair the quality of life (QoL) of patients (Schrag et al., 2000) and caregivers (Schrag et al., 2006). mNCDs in PD include fronto-striatal syndrome due to dopaminergic shortage and include deficits of executive functions, such as planning, mental flexibility and working memory (Kehagia et al., 2010; Dirnberger and Jahanshahi, 2013). As the disease progresses, cognitive deficits spread into other cognitive domains and may deteriorate into major Neurocognitive Disorders interfering with independence in everyday activities (Litvan et al., 2011).

To date, in patients with idiopathic PD three studies have evaluated the efficacy of tDCS on executive functions. Boggio et al. (2006) investigated tDCS effects on 18 patients (mean AGE = 61 45–71; mean MMSE = 24.4) diagnosed idiopathic PD using a three-back working memory task. Patients performed the task during anodal tDCS (A-tDCS) on left dorsolateral prefrontal cortex (L-DLPFC), A-tDCS on motor cortex (M1) and sham. In addition, the authors tested whether the effects depended on the intensity of stimulation; performing a control experiment with different intensities a constant current of 1 mA or 2 mA that was applied for 20 min. The authors found that after a single session of 2 mA A-tDCS over the L-DLPFC patients improved in the accuracy of the 3-back memory task. The other stimulation conditions (sham, 1 mA A-tDCS on L-DLPFC or A-tDCS on M1) were not effective. Their results were recently reinforced by a controlled cross-over, tDCS combined fMRI single session study of Pereira and colleagues. In this study (Pereira et al., 2013) 16 patients (mean AGE = 61.5 ± 0.9; mean MMSE = 27.7) diagnosed as idiopathic PD were randomized to receive A-tDCS on L-DLPFC (F3) or A-tDCS on L-TPC (P3-T5) and immediately after performed a verbal fluency task inside the scanner. The authors found an improvement on the phonemic fluency task after a single session A-tDCS over the L-DLPFC. Furthermore, fMRI analysis of connectivity demonstrated that A-tDCS applied over the L-DLPFC produced a greater activation of the specific functional networks engaged by the task compared to A-tDCS over temporo parietal cortex TPC. While these two studies demonstrated that tDCS may improve specific components of executive function, the effects were short-lasting and did not generalize to everyday functioning. A subsequent multicenter study then investigated the efficacy of a multiple sessions protocol in idiopathic PD patients on multiple cognitive domains including executive function, attention, perceptual-motor abilities, learning and memory. Here, 10 consecutive sessions (over 2 weeks) of A-tDCS over L-DLPFC or A-tDCS over R-DLPFC or sham, were administered by a randomized between subject design on 18 patients (6 in each group). Cognitive functions were evaluated before, at the end of stimulation sessions and at 1 month follow-up. It was found A-tDCS over both the L and R-DLPFC compared to sham improved performance only on Trial Making Test B at the 1-month follow-up but not on the other outcome measures.

Overall, these studies demonstrate that A-tDCS over the prefrontal cortex may be effective for improving executive functions, but it must be emphasized that these studies lack sufficient numbers of patients, statistical power and more importantly transfer of benefits into everyday functioning. Across the studies, there is a general agreement on the parameters of stimulation. While the positions of active electrode A- L-DLPFC(F3) and reference contralateral supraorbital and also the intensity (2 mA) and the duration (20 min) of stimulation are the same or similar in all studies, it is not clear what the criteria are for selection of the outcome criteria, such as reliability or validity. Furthermore, it is unclear what the most sensitive test to measure tDCS efficacy on cognitive domains may be. In sum, this evidence encourages and warrants further investigation. In future studies shared methodology are necessary to allow comparison across studies and to assert the usefulness of tDCS for cognitive rehabilitation in PD.

### Alzheimer's disease (AD)

Alzheimer's disease (AD) is a progressive disease that arises on a neuropathological background of amyloid plaques (APs) and neurofibrillary tangles (NFTs). AD is the most common form of major NCD, where symptoms gradually progress over a number of years with memory loss and decline of intellectual abilities serious enough to interfere with daily life. This disturbance is related to the degree of brain atrophy in medial temporal lobe involving entorhinal cortex and hippocampus, and also prefrontal areas. Memory disturbances appear early, at first affecting the ability to learn and retrieve information, and later causing impairments in recognition memory and attention. Ferrucci et al. (2008) in a randomized cross-over study tested 10 AD patients (mean AGE = 75.2 years; MMSE = 22.7 overlapping 1.8) on recognition memory and visual attention. Patients underwent a single session protocol of A-tDCS or C-tDCS or sham over bilateral temporo-parietal areas (two electrodes on the scalp and one reference on deltoid). Before and 30 min after stimulation patients performed a word recognition test and a visual attention test. It was found that A-tDCS increased accuracy in word recognition memory, and conversely C-tDCS decreased accuracy. Performance on visual attention did not change. A successive randomized cross over single session study of Boggio et al. (2008) assessed the efficacy of A-tDCS on recognition memory, working memory and attention in 10 AD patients (MMSE between 12 and 25). Patients participated in three separate sessions to receive A-tDCS over left temporal cortex (L-TC) or A-tDCS over the L-DLPFC or sham. For all conditions, the reference, cathode electrode (35 cm^2^) was placed over the right supraorbital area. Stimulation was delivered during a Visual Recognition Memory task, Stroop, or Digit Span task, with the order randomized across participants. Tasks started 10 min after stimulation onset and lasted until the end of stimulation. Each condition was separated by at least 48 h. It was found that both A-tDCS over temporal or prefrontal cortex improved Visual Recognition Memory performance compared to sham. Attentional performance measured by the Stroop was unchanged. Albeit these two studies showed that A-tDCS may positively modulate aspects of memory, the effects were small and without any follow-up measures. To overcome these limitations, 3 years later, Boggio and colleagues performed a multicenter, cross-over multiple sessions follow-up study. Here, fifteen AD patients underwent five consecutive A-tDCS over L-TPC and R-TPC bilaterally or sham. Visual Recognition Memory, visual attention and general cognition (MMSE) were assessed before, immediately after the end of stimulation sessions and at 4 weeks follow-up. They found that A-tDCS patients improved on Visual Recognition Memory compared to sham. Moreover, these effect persisted 4 weeks after the end of stimulation. There were no changes in visual attention or general cognition.

To date, two studies have assessed the combined use of tDCS and cognitive training. Cotelli et al. (2014) evaluated for the first time the impact of tDCS combined with individualized associative memory training (iMT-FNAT) on specific associative memory test and learning and memory, attention, language and perceptual-motor domains. Here, 10 consecutive sessions (over 2 weeks) of A-tDCS over the L-DLPFC during iMT or A-tDCS over the L-DLPFC during motor training or sham tDCS during iMT; were administered in a randomized between subject design in 36 patients (12 in each group). Neuropsychological assessment and Face-Name Association memory Task (FNAT) were completed at 4 time points (before, 2 weeks after, 3 and 6 months after). An improvement only in selectively trained stimuli induced by iMT irrespective of site by both A-tDCS and sham tDCS group was found. In other words A-tDCS over the L-DLPFC did not have an additive effect on the FNAT computerized training. Moreover, the improvement was task-stimuli specific and did not generalize to other domains. In a subsequent single case study Pennolazzi and colleagues examined the effectiveness of tDCS combined with Individualized Computerized Task (iCT) performance (Penolazzi et al., 2014). An AD patient of 60 years (MMSE 23) underwent 10 sessions A-tDCS over the L-DLPFC followed by iCT. iCT (based on the patient's impairment) included verbal working memory task, phonemic fluency task and continuous performance task. Effects on cognitive performance were evaluated by the iCT and by extensive neuropsychological assessment of global cognitive functioning. The authors found iCT combined with anodal stimulation to be better than iCT combined with the sham. Thus, combined 10 daily sessions of A-tDCS over the left prefrontal cortex and iCT slowed down the cognitive decline of the patient more than iCT alone.

The differences in the latter two studies (Cotelli et al., 2014; Penolazzi et al., 2014) may emerge from the key methodological variations between them such as training during stimulation (Cotelli) or training follow stimulation (Penolazzi). Moreover, the authors utilized diverse cognitive training together with different outcome measures to assess stimulation effects. In addition Cotelli et al. used an extra-cephalic reference and Penolazzi et al. a cephalic reference which will have resulted in a different current flow.

Recently there have been two studies with a larger number of patients than previous studies. Suemoto et al. (2014) examined the efficacy of A-tDCS in 40 moderately cognitively impaired AD patients (MMSE 10–20) for apathy and global cognitive functioning. Here, six sessions of A-tDCS on L-DLPFC, vs. sham, were administered in a randomized cross-over design. Patients were evaluated at baseline, after the first and the second week of stimulation, and after 1 week without intervention. The authors found that A-tDCS had no effect on apathy or on global cognitive performance, or the ADAS-Cog sub-items. This study shows that repeated A-tDCS over the left prefrontal cortex in patients with a state of relatively advanced deterioration is not able to improve their cognitive deficits or apathy. In a multiple session, 2 months follow up study of Khedr et al. (2014) 34 patients (mean AGE = 69.7 years; mean MMSE = 18.1 range 12–23) were tested. Here, ten sessions of A-tDCS or C-tDCS over the L-DLPFC, vs. sham, were administered in a randomized between subjects study design. Global cognitive functioning (MMSE) and Intelligence (WAIS-III) were assessed at four time points (baseline; end of the 10 sessions; 1 and 2 months after the end). Furthermore, motor cortical excitability and the P300 event-related potential were assessed at baseline and after the last tDCS session. The authors found that 10 sessions of both A-tDCS or C-tDCS over the L-DLPFC improved MMSE compared to sham with a further increase at 1 and 2 months follow-up. Only C-tDCS seemed to have a minor positive effect on a subscale of the WAIS-III.

To sum up, there is some evidence from randomized controlled clinical studies showing a beneficial effect of A-tDCS on some specific components of memory. However, it is evident that there is a great deal of methodological heterogeneity across these studies. First, there are diverse stimulation protocols adopted, only two studies used the same location and size of the electrodes (Boggio et al., 2008; Suemoto et al., 2014). Additionally, some studies preferred an extra-cephalic reference to avoid unwelcome interference effects from brain areas underlying the reference electrode. In general, a better definition of stimulation protocols needs to be provided. Second, most of these studies do not consider the fact that cognitively impaired patients can be highly variable in the manifestation of their cognitive problems and in some cases group variability between patients and within a patient from 1 day to the next can mask the effectiveness of a treatment. Third, by and large most studies did not measure whether the improvement in a specific task has generalized to everyday life. Indeed it is imperative to discriminate between increase in performance on a specific cognitive task and recovery in more general daily life activities demanding that cognitive function. Further studies should consider the individual characteristics of each patient, better define stimulation parameters and outcome measures and look at translation into everyday cognitive functioning.

### Unilateral spatial neglect

Unilateral spatial neglect is a neurological syndrome that develops following damage to one hemisphere of the brain. It is characterized by a deficit in attention to and awareness of one side of space. It is defined by the inability of a person to process and perceive stimuli on one side of the body or environment, where that inability is not due to a lack of sensation. Unilateral spatial neglect results most commonly from brain injury to the right cerebral hemisphere, causing visual neglect of the left-hand side of space.

Overall, the rational for the studies using tCDS in patients with unilateral neglect is based on Kinsbourne's interhemispheric conflict model. According to this model parietal lobes may exercise interhemispheric inhibition through the connections of the corpus callosum balancing allocation of visuospatial attention toward both hemifields. Brain lesions, as a result of stroke, damage this balance. For this reason A-tDCS is applied to the lesioned hemisphere to increase cortical excitability and the C-tDCS to inhibit the over-activated unlesioned hemisphere.

In a double-blind, crossover, controlled experiment Ko et al. (2008) enrolled 15 right-handed subacute stroke patients (mean Age = 62.1 ± 8.8 years; mean time post-onset = 29–99 days) with left visuospatial neglect due to right-sided cortical and/or subcortical vascular lesions. Patients participated in a single session protocol of A-tDCS over the right parietal cortex (R-PC) (damaged hemisphere). Before and after “treatment” patients performed a line bisection test and a cancelation test. The authors found an improvement of performance in both tests, indicating a recovery of neglect symptoms, compared to sham. Sparing and colleagues in a randomized cross-over study (Sparing et al., 2009) tested 10 right-handed patients (mean age = 57.3 years; mean time post-onset 2.9–3.5 months) with left visuospatial neglect due to right-sided vascular lesions. Here, a single session of A-tDCS over the right posterior parietal cortex (R-PPC; damaged hemisphere) or C-tDCS over the left posterior parietal cortex (L-PPC) were conducted. A visual search task and a computerized Line Bisection task were administered before and after tDCS. The authors found that both C-tDCS over the undamaged PPC A-tDCS over the damaged PPC reduced symptoms of visuospatial neglect. More recently, a rather unconventional protocol was pursued by Sunwoo et al. (2013) [14], who used two stimulators and four electrodes on the scalp. A double-blind randomized cross-over study was performed to assess the impact of dual-mode montage with A-tDCS over the R-PPC(P4) and C-tDCS over the L-PPC(P3) concurrently, and to compare single-mode A-tDCS over the R-PPC alone and sham on 10 patients with chronic stroke induced neglect (mean age = 62.6 years ± 13.3 mean time post-onset 27.8 ± 60.4 months). Before and after “treatment” patients performed a line bisection test and cancelation test. It was found that both dual-mode and single-mode tDCS were safe and beneficial for neglect symptoms.

Two studies assessed the impact of multiple sessions of tDCS on Neglect patients. A combined approach was followed by Brem et al. (2014), who combined tDCS and cognitive training. Here, five consecutive sessions of ordinary neglect therapy combined with biparietal A-tDCS over the R-PPC and C-tDCS over the L-PPC, vs. sham, were administered in a double-blind, single case cross-over design in a 72-year-old, ambidextrous male patient with stroke of the right posterior cerebral artery. Neuropsychological assessment before and after treatment were evaluated by Test for Attentional Performance (TAP) (which includes covert attention, alertness, visual field) and the Neglect-Test (NET) (line bisection, cancelation, copying). Furthermore, generalization on activities of daily living (ADL) was also evaluated. It was found that with bilaterally active PPC tDCS improvement was significantly higher than during standard neglect therapy alone or sham. The authors highlighted for the first time the additive effects of tDCS and standard neglect therapy on functional improvement. Importantly the beneficial effects of tDCS was maintained over a follow-up period of 1 week and 3 months. A subsequent study by Smit et al. (2015) evaluated the immediate and long-term effects of multiple sessions of tDCS on five severe chronic hemispatial neglect patients. Here, five consecutive sessions of bilateral A-tDCS over the R-PPC and C-tDCS over the L-PPC, vs. sham, were conducted in a randomized double-blind cross-over design. Neuropsychological assessment before and after treatment by Behavioral Attention Test (BIT) indicated no symptomatic improvement after bilaterally PPC tDCS stimulation. While these two studies examined the effects of multiple sessions of tDCS, Brem and colleagues tested a single stroke patient in the subacute phase, while Smit and colleagues tested five stroke patients in the chronic phase.

In summary, these results are encouraging, but further clinical trials with larger number of patients and follow up are needed. Moreover, translation of symptoms amelioration into everyday activities need to be measured.

### Aphasia

Aphasia is an impairment of language, affecting the production or comprehension of speech and the ability to read or write. Aphasia is always due to injury to the brain most commonly from a stroke, particularly in older individuals. Aphasia can be so severe as to make communication with the patient almost impossible, or it can be very mild. It may affect mainly a single aspect of language use, such as the ability to retrieve the names of objects, or the ability to put words together into sentences, or the ability to read. Generally multiple aspects of communication are impaired. In this form of aphasia, speech output is severely reduced and is limited mainly to short utterances of less than four words. Vocabulary access is limited and the formation of sounds by individuals with Broca's aphasia is often laborious and clumsy. The person may understand speech relatively well and be able to read, but be limited in writing. Broca's aphasia is often referred to as a ‘non fluent aphasia’ because of the halting and effortful quality of speech.

In patients who suffer from non-fluent aphasia the studies so far evaluated the immediate effect of tDCS on naming abilities.

The first study was conducted by Monti et al. (2008), who included eight right-handed chronic non-fluent aphasic patients in a randomized controlled cross-over study. They tested the effect of A-tDCS or C-tDCS over the left Broca's area (damaged hemisphere; crossing point between T3-Fz and F7- Cz) and sham on picture naming task accuracy. An improvement in accuracy after C-tDCS compared to A-tDCS and sham was found. It is worth noting that this study is not in line with the Neglect studies cited above in which A-tDCS was applied over the damaged hemisphere and C-tDCS over the intact hemisphere. Even so these study are difficult to compare because of the differences in the parameters adopted.

Subsequent studies evaluated the effect of A-tDCS over the left damaged hemisphere during naming training in post-stroke non-fluent aphasia patients on naming task accuracy with mixed evidence.

Fiori et al. (2011) tested three aphasic patients with anomic difficulties using a picture-naming task. In a randomized double-blind cross-over study, they administered five consecutive sessions of A-tDCS over the Wernicke's area (CP5), vs. sham applied during intensive anomia training. The authors found a significant improvement in the picture-naming task accuracy.

More recently, in eight stroke patients with distinct types of aphasia, Volpato et al. (2013) examined the effect of A-tDCS on naming abilities. Here, ten consecutive sessions over 2 weeks of A-tDCS over the L-Broca's area, vs. sham were administered in a randomized cross-over design. The authors found no significant differences between A-tDCS and sham on naming abilities. Similarly, in a randomized between subjects study, Polanowska et al. (2013) conducted 15 sessions of A-tDCS over L-Broca's area followed by language training. Patients were assessed by Boston Diagnostic Aphasia Examination before, immediately after treatment and at 3 months follow up. Again, the authors found no significant differences between A-tDCS and sham groups. In another small sample study, three patients with chronic stroke, in a cross-over design, received naming training during A-tDCS over the left frontal perilesional areas vs. sham. Vestito et al. (2014) found that naming abilities, as assessed by a computerized naming task, improved in the A-tDCS group compared to the sham group. A rather unconventional protocol was followed by Lee et al. who simultaneously used two stimulators. A randomized cross-over study were performed to assess the impact of a dual-mode montage with A-tDCS over the L-IFG(F7) and C-tDCS over the R-IFG(F8) concurrently, compared to single-mode A-tDCS over the L-IFG alone and sham on 11 patients with chronic stroke-induced aphasia. During the last 15 min of tDCS, speech therapy was provided. Before and after treatment, patients performed a picture naming test and a picture description test. It was found that both dual-mode and single-mode tDCS improved naming accuracy and reaction times compared to sham. More recently, Wu et al. (2015) examined 12 sub-acute stroke patients with aphasia using a picture naming task and an auditory picture identification task. Moreover, they measured cortical excitability by electroencephalography (EEG) nonlinear dynamics analysis. In a randomized controlled cross-over study they administered A-tDCS over the L-posterior perisylvian region vs. sham and patients received 20 sessions of speech therapy. The authors found an improvement in picture naming and auditory comprehension after A-tDCS compared with sham. Furthermore, EEG analysis indicated that naming improvement correlated with higher activation in the brain language network.

Two other studies used an innovative approach to position the electrodes. Baker et al. (2010) in a randomized controlled cross-over study tested 10 patients in the chronic phase with mild to moderate post stroke non-fluent aphasia. They administered five consecutive sessions of A-tDCS over the left frontal cortex vs. sham during computerized anomia training. Each patient performed a naming task inside the scanner. Then fMRI results for each individual was used to place the electrodes. A significant improvement in naming accuracy after A-tDCS compared to sham was reported. The improvement was maintained 1 week after treatment. In a subsequent study Fridriksson et al. (2011) tested eight patients with stroke-induced fluent aphasia utilizing the same picture naming task and electrodes placement procedure. Here, five consecutive sessions of A-tDCS vs. sham were administered in a randomized controlled cross-over design. Reduced RTs during naming were also found after A-tDCS which was maintained after 3 weeks.

Some studies assessed the long-term therapeutic benefits of tDCS on naming. In the chronic stage, Marangolo et al. (2014) included seven patients with stroke-induced non-fluent aphasia in a randomized controlled cross-over study. They administered five consecutive sessions of A-tDCS over the L-Wernicke's area or L-Broca's area vs. sham during training for action naming. Training consisted of three groups of video clips representing actions that patients had to name. Naming accuracy was assessed before treatment, immediately after and at 1 and 2 weeks follow-up. The authors found significantly improved accuracy after A-tDCS over the Broca's area compared to Wernicke's area and sham. The effect persisted at 4 weeks follow-up. This result highlights the functional importance of Broca's area in verb processing. Manenti et al. (2015) included one chronic stroke patient with non-fluent aphasia in a pretest posttest design study without sham control. Here, twenty consecutive sessions of bi-hemispheric A-tDCS over the L-DLPFC and C-tDCS on R-DLPFC were followed by individualized verb anomia training. An extensive language evaluation was completed before, after treatment and at 12, 24, and 48 weeks after. The authors found an improvement in verb naming and a decrease in self-perceived difficulties in social situations and improved linguistic abilities suggesting an impact of the treatment on the daily life of the patient. Importantly, the authors asserted that this effect persisted 48 weeks after stimulation.

Two studies attempted to improve aphasia symptoms by stimulating the right hemispheric homolog areas. In the chronic stage, Flöel et al. (2011) included 12 patients with moderate to severe aphasia in a randomized controlled cross-over study. Here, A-tDCS or C-tDCS over the R-temporo parietal cortex (R-TPC) vs. sham combined with anomia training were conducted. The authors found that A-tDCS significantly enhanced the overall training effects compared to sham and the effect persisted after 2 weeks. Similarly, in a randomized controlled cross-over study, Vines et al. (2011) enrolled six patients with moderate to severe aphasia. They used A-tDCS over the right inferior frontal gyrus (R-IFG) during melodic intonation therapy (MIT) for three consecutive days. They reported that combining A-tDCS with MIT significantly improved verbal fluency compared to sham with MIT.

Other studies attempted to restore language abilities by suppressing the right homolog language areas with C-tDCS. In the sub-acute stage, You et al. (2011) included 21 patients with comprehension impairment in a randomized controlled between subjects design. Here, ten sessions of conventional speech therapy were combined with A-tDCS over the left superior temporal gyrus or C-tDCS over the right superior temporal gyrus or sham. It was found that auditory verbal comprehension improved after C-tDCS over the right hemisphere compared to A-tDCS and sham. Similarly, in a double-blind randomized controlled study, Kang et al. found that five consecutive sessions of C-tDCS over the R-Broca's area combined with word-retrieval training improved performance in picture-word matching task.

Three studies concentrated on factors associated with response to C-tDCS protocol. Jung et al. (2011) included 37 stroke patients from acute to chronic in a pretest posttest design study without sham control group. Here, ten consecutive sessions of C-tDCS over the R-inferio frontal gyrus were administered. The authors assessed the effect of tDCS by the Korean version of Western aphasia Battery. Using regression statistical models it was found recovery after C-tDCS was more in patients with less severe aphasia who had started “treatment” within the first months after stroke. In a more recent, randomized controlled cross-over study, Rosso et al. (2014) adopted an innovative fMRI combined tDCS approach looking for inter-individual variability. They found C-tDCS over the R-Brocas's area improved performance on a computerized picture naming task. More importantly the authors found that improvements in naming after C-tDCS of the R-Broca's area relies on several structural and functional factors.

One study assessed the efficacy of an individualized tDCS treatment in stroke-induced non fluent aphasia in chronic patients. Shah-Basak et al. (2015) ingeniously took into account the individual variability in response to tDCS. In the first phase of the study the authors individualized the protocol based on individual responses to the A-tDCS or C-tDCS over the L-IFG or R-IFG. Then in a randomized cross-over study, 10 sessions of active tDCS or sham were administered during a picture naming task. Language abilities were assessed before, after treatment, 2 weeks and 2 months after. Aphasia symptoms improved after the active tDCS treatment compared to sham and the improvement remained 2 month after the end of treatment. This study suggests that an individualized protocol may be effective in improving stroke-induced chronic aphasia symptoms overcoming the high variability between patients.

An unusual approach was followed by Santos et al. (2013). They included nine teenaged chronic stroke sufferers from non–fluent aphasia in a pretest posttest design study without sham control group. Here, ten consecutive sessions of A-tDCS over the primary motor cortex (M1) of the healthy hemisphere were administered. Language level was assessed before and immediately after the treatment. They found a significantly improved performance in sentence comprehension, naming and specific animal name category verbal fluency.

In sum, there are some randomized controlled evidence that indicated a favorable effect of tDCS in improving language symptoms related to aphasia. Again, there is a great deal of methodological heterogeneity across these studies. Various approaches have been undertaken including the application of A-tDCS over the left damaged hemisphere concomitant to a naming training or to restore naming abilities by suppressing the activation of the right homolog language areas with C-tDCS. In a rather original fashion, one study took individual differences in response of tDCS into account.

## Methodological issues

### Clinical and demographic characteristics of samples

It is important to remember that neurodegeneration or insult or injury to the brain does not affect two people identically. Such individual differences also lead to differences in the evolution of the disease. Even though patients have been diagnosed with the same disorder there are substantial differences between them. In the case of progressive degenerative diseases such as PD and AD, the evolution and progression of the disease is unique in each case and each person responds differently to treatment.

Furthermore, numerous studies have argued that there are some important factors that can affect the evolution of NCD. Cognitive Reserve (CR), for instance, is a factor that would be reasonable to consider in the case of neurodegenerative disorders (Stern, 2002). CR is a term describing the resilience of the brain following the brain damage. CR is defined as the ability to optimize or maximize performance through differential recruitment of brain networks (Scarmeas et al., 2003). It depends on factors such as education, profession, lifestyle and leisure activities which play an important role in determining how many alternative resources are available to be used to compensate for the cognitive deficits.

With regards to medical conditions that occur after a brain injury such as unilateral spatial neglect and aphasia there are many points to consider. First, it is almost impossible to find two patients with damage that affects exactly the same part of the brain because of anatomical differences between individuals. Cerebral infarction and hemorrhage may be more or less circumscribed involving diverse brain areas. Second, even if we find two patients with exactly the same injury the two individuals could have a different ability to recover or to compensate. Third, in patients who have suffered a stroke an important aspect to consider is whether patients are treated in the subacute phase (within 6 months) or in the chronic phase. It has been suggested that the brain is more sensitive to reorganization during the months immediately after the stroke. Fourth, it would be important to consider the pre-morbid cognitive state of the participants.

Selection of patients for inclusion in the experimental group is an important and sometimes difficult process in this areas of research. Group variability can affect the outcome of a study. It is extremely important to minimize the heterogeneity of patients in order to gain a better understanding of tDCS as a therapeutic technique. Bearing this in mind, there are remarkable differences in the demographic and clinical characteristics of patients undergoing tDCS treatment in the studies examined (see Table 2). For example, regarding AD in the study of Boggio et al. (2008) there is a huge intragroup variability. A patient with an MMSE score of 12 (moderate cognitive impairment) is in the same group as a patient with an MMSE score of 25 (mild cognitive impairment). These patients were comparable for age, respectively 85 and 89, but different for years of education, respectively 4 and 11 years. Suemoto and colleagues recruited patients and divided them into two groups with mean ages of 79.4 and 81.6 years; 5 and 4.5 years of education and a MMSE score of 15 and 15.4 (Suemoto et al., 2014); while in the single case study of Penolazzi et al the patient's age was 60 years, with 18 years of education and an MMSE of 23 (Penolazzi et al., 2014). In the study of Khedr et al. (2014) the average age of the three groups of patients recruited was 68.5, 70.7, 67.3 years and MMSE scores of 18.4, 18.8, and 16.9; and years of education was not reported. Regarding PD, Boggio et al. (2006) recruited patients with a score of 36.8 for Experiment 1 and 43 for Experiment 2 on the UPDRS while in the study of Pereira et al. (2013) patients were recruited with a mean score of 13.3 on the UPDRS. Furthermore, in the study of Boggio et al the average years of education of the patients was 4.7 years for Experiment 1 and 5.3 years for Experiment 2; while in the study of Pereira et al the patients' average schooling was 12.3 years. With regards to unilateral spatial neglect, there are remarkable intragroup differences in the site of damage of the patients. In the studies reviewed in the same experimental group there are patients with damage limited to the basal ganglia, patients with more extensive lesions covering frontal, temporal and parietal lobes or frontal parietal occipital lobes. Another factor on which the patients differed is the duration of illness post onset. Most of the studies recruited patients in the subacute phase within 6 months after stroke (Ko et al., 2008; Sparing et al., 2009; Brem et al., 2014). Only two studies enrolled patients in the chronic phase (Sunwoo et al., 2013; Smit et al., 2015).

In the existing studies, it is often neglected that clinical features of patients may affect the outcome of tDCS. To date, little importance has been given to patient characteristics which could in part explain the variability in the response to the tDCS. Future studies should try to control as much as possible factors that may influence the outcome of therapeutic application of tDCS in cognitive rehabilitation.

### tDCS parameters, electric fields and neuroanatomy

tDCS scalp surface anodal and cathodal electrodes inject low amplitude direct currents (0.5–2 mA) through the head and these currents are applied from few seconds to several minutes. This results in an electric field and a current density generated in the scalp and brain. In the studies which first measured the impact of this electric field on the human brain, tDCS was combined with TMS to investigate modification of cortical excitability. The first study to explore cortical excitability investigated the effects of up to 0.5 mA currents applied using an M1-chin montage on the size of the motor evoked potential (MEP) (Priori et al., 1998). However, the first “modern” study to use the standard current and electrode parameters was published 2 years later (Nitsche and Paulus, 2000).

Generally if the anode is placed above the motor cortex, after DC stimulation, single pulse TMS will result in a larger MEP (Day et al., 1987; Rothwell, 1997). If the cathode is placed over the motor cortex, the MEP size will be reduced. Thus, long-lasting and polarity-dependent changes in neural excitability of the human cortex are elicited. This effect is conceivably due to depolarization of somatic membrane potentials by anodal currents and hyperpolarization of soma by cathodal currents, as observed in animal studies (Bindman et al., 1964).

Several studies have been performed in humans in order to understand the physiological mechanisms of tDCS. It has been shown that the effects on the MEP can be modified, prolonged or even reversed by drugs acting on the central nervous system (Stagg and Nitsche, 2011). Importantly, it seems that neuroplastic after-effects of tDCS are NMDA-receptor dependent (Liebetanz et al., 2002). Moreover, anodal after-effects can be selectively suppressed by both the sodium channel blocker carbamazepine and the calcium channel blocker flunarizine (Nitsche et al., 2003). These studies demonstrated that is possible to measure in humans the effects of direct current application by TMS at the motor cortex.

Based upon what is known about the process of MEP production a growing interest for examining the anodal and cathodal tDCS effects on other brain areas has emerged. It is worth noting that it is absolutely unclear whether it is possible to generalize these processes in the modification of MEPs to other more complex cognitive processes. In spite of this during the last decade a considerable amount of literature has been published on the capacity of tDCS to alter human brain functions over numerous brain areas and in the treatment of a wide range of diseases. This interest has been facilitated by the fact that from a neuroscience point of view, the causal and interventional nature of tDCS is particularly exciting. This exponential growth of published works is somewhat surprising if we consider that the understanding of the basic principles of tDCS have not yet been achieved. Perceiving, remembering, reasoning and language are more complex processes than MEPs. Moreover, many studies are based on the theoretical assumption that placing the anode electrode over the area of interest would enhance precisely the activity of the target region and conversely placing the cathode would decrease the activity, which raises a number of problematic points. One problem with this approach is the low spatial resolution of tDCS. The rationale that putting an electrode on the scalp over a region of the brain results in a precise stimulation of that region, and only the target region is unlikely to be accurate. Indeed the major drawback is that the amount and distribution of current flow fluctuates extensively as a function of individual physiology and anatomy. So investigators who use tDCS are not in a position to make accurate inferences about the operation of a specific brain area. It is not sufficient to only examine the behavioral outcome to ascertain the specific involvement of a brain area and rule out the possible role of another area.

It therefore follows that an urgent question that needs to be asked is how the current is distributed in the brain during tDCS. To answer this question recently modern mathematical models that integrate structural resonance magnetic images (MRI), have been developed to understand the distribution of the electric field in the brain (Bikson et al., 2012; Datta et al., 2012). These modeling approaches showed that the effects of administering a current in the brain using a particular configuration of the electrodes are the result of many factors such as the spatial distribution of the electric field induced in the gray matter (GM) and white matter (WM), the orientation of the electric field relative to the neurons and many other factors (Miranda et al., 2013).

In light of this complexity, the application of tDCS to neurocognitive disorders should consider the brain morphological heterogeneity of patients. Along these lines it is difficult to conceive that the same stimulation protocols with the same parameters of stimulation may be optimal in different patients. For instance, in the case of degenerative disorders characterized by marked atrophy such as AD it is difficult to conceive that the same dose of tDCS is optimal in two different patients as suggested by Mahdavi et al. (2014). An interesting parallel in this regard is with deep brain stimulation (DBS). DBS is a neurosurgical procedure in which an electrode is implanted in the brain and is controlled by a neurostimulator. In DBS the patient's behavioral state is used as an indicator of how to change the parameters. That is to say that the frequency, pulse width and voltage of stimulation are adjusted based on the positive response of the symptoms of each patient and simultaneous avoidance of side-effects (Kringelbach et al., 2007).

**Figure 3:**
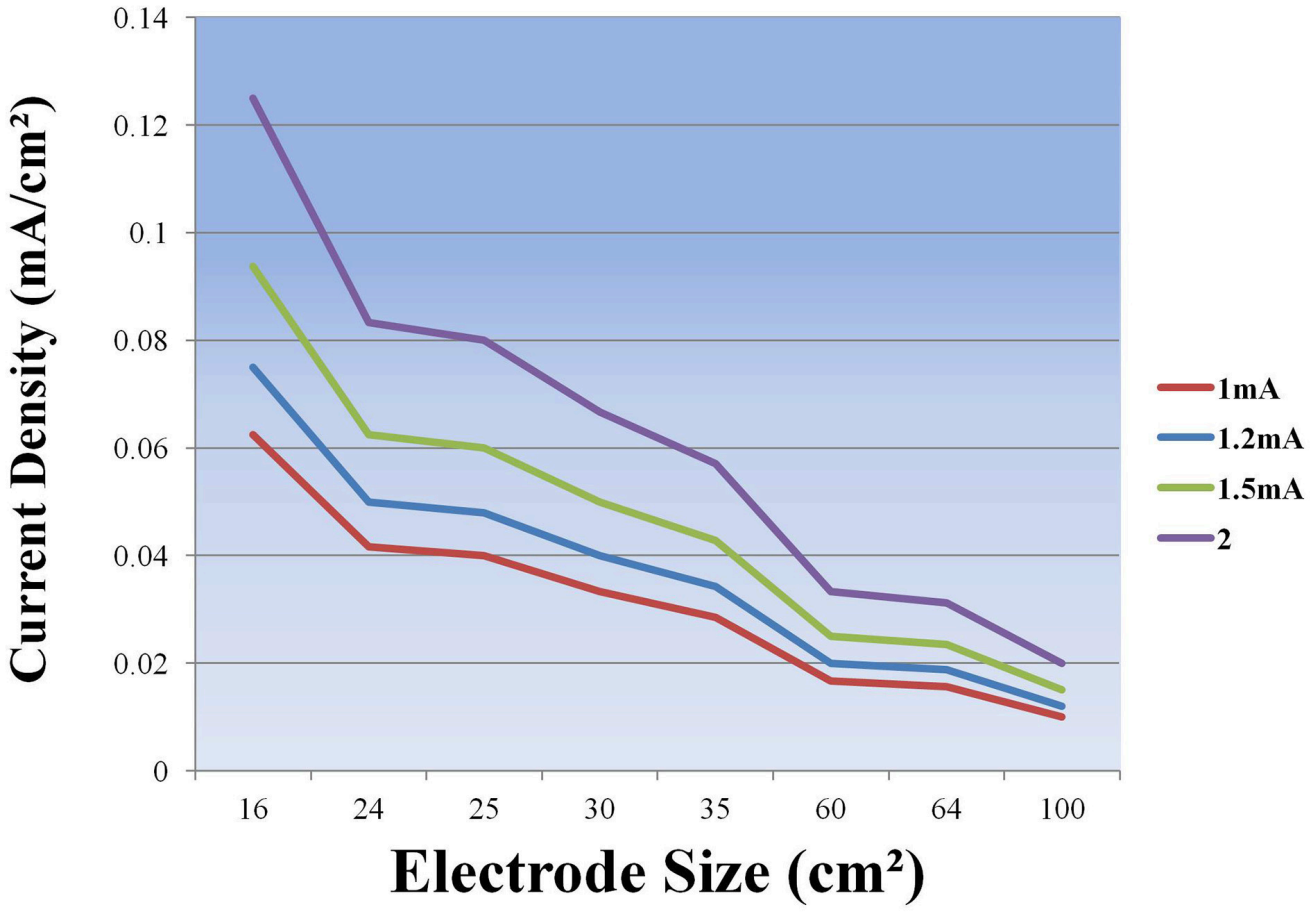
**Current Density (mA/cm^**2**^) as a function of electrode size**.

It is evident that tDCS of both the normal and the diseased brain depends on a number of factors such as the stimulation parameters including the electrode localization, duration and intensity (see Table 3 and Figure 3) of stimulation and also the patient characteristics such as age, disease stage, years of education and premorbid level of functioning which influence cognitive reserve. The studies we reviewed above show remarkable differences regarding the criteria for selecting the patients, the placement of the electrodes, the duration and intensity of stimulation (see Tables 1, 2) and this makes it very difficult to compare the results across studies. More research into the complex dynamics of the current flow it is essential before obtaining a definitive optimization of stimulation protocols (see de Berker et al., 2013).

**Table 3:** **Current Density (mA/cm^**2**^) of different electrode dimensions**.

**Max Current Intensity (mA)**				
**Electrode size (cm**^2^**)**	**1**	**1,2**	**1,5**	**2**
16	0.063	0.075	0.094	0.130
24	0.042	0.050	0.062	0.083
25	0.040	0.048	0.060	0.080
30	0.033	0.040	0.050	0.067
35	0.029	0.034	0.043	0.057
60	0.017	0.020	0.025	0.033
64	0.016	0.019	0.023	0.031
100	0.010	0.012	0.015	0.020

Further research in this area may include an integration of data coming from other techniques such as functional magnetic resonance imaging (fMRI) and magnetoenchephalografy (MEG). In the coming years, it is important to work toward optimizing tDCS protocols for cognitive rehabilitation based on the initial response of each patient to this therapeutic application.

### State of the brain during stimulation

An important and fundamental question that remains to be addressed is “Why does depolarizing cells by administering a very weak current in the brain modify elaborate cognitive processes?” The theoretical model that may be relevant to answering this complex question is stochastic resonance (SR). SR has been observed throughout nature and it has been reported in physiological neural populations and networks (McDonnell and Ward, 2011). “*SR is observed when noise added to a system changes the system's behavior. Stochastic resonance (SR) is a phenomenon in which a signal that is normally too weak to be detected by a sensor, can be enhanced by adding white noise to the signal, which contains a spectrum of frequencies. A proportioned amount of added noise results in the maximum enhancement a disproportionate noise intensity degrade detectability or information”* (Moss, 2004).

Along similar lines, conceptualizing the administration of tDCS as adding noise to the brain system, one can argue that when a proportionate amount of noise enters the system it would maximize behavioral performance, and conversely if disproportionate noise enters the system it would not produce any effect or worse behavioral performance. This model seems appropriate to explain the high variability in the reported effects of tDCS (Jacobson et al., 2011; Horvath et al., 2015). The implication of the SR model is that the activity status of the system is important. In this case the system is the brain. It follows that the activity of the brain during tDCS is extremely important in determining the overall effect of the stimulation as previously suggested by Silvanto et al. (2008) and more recently by Miniussi et al. (2013). First, a critical factor which is necessary to consider is whether stimulation should be applied during behavioral / cognitive treatment or whether stimulation should be applied offline. Second, following the SR model, it is necessary to consider how many sessions are needed to change the behavior of the “brain system.” Third, not only the timing of stimulation and the number of sessions but also the difficulty of the task or training must be considered. Depending on the level of difficulty of the task that the patients have to engage in, more or less cognitive resources would be required, which is also an important variable. Fourth, it is extremely important to determine whether any improvement generalized on untrained cognitive tasks. Evidences indicates that cognitive enhancement can occur at the expense of other cognitive functions (Iuculano and Cohen Kadosh, 2013). To our knowledge very few publications in the literature have also measured other cognitive domains (different from that central for the study) to control for possible cognitive side effects. Future studies should consider all these factors for a more effective therapeutic protocol.

## General conclusions

The present review considered the application of tDCS for the cognitive rehabilitation of four neurocognitive disorders: Parkinson's Disease, Alzheimer's Disease, Unilateral Hemispatial Neglect and Aphasia. While in PD there is a general agreement on the parameters of stimulation, what might constitute the most sensitive test to measure t-DCS efficacy on cognitive domains remains unclear. By contrast, for AD, unilateral neglect and aphasia, the variability across studies in the stimulation parameters used, the target site of tDCS stimulation and on the intensity of the stimulation, makes drawing firm conclusions about efficacy more difficult.

Nevertheless, most of the studies reviewed reported a positive effect of tDCS in all these neurocognitive disorders. However, in cognitive rehabilitation it is critical to move beyond statistical significance and consider clinical significance of effects. Such positive evidence of tDCS-induced cognitive benefit cannot be considered as fully reliable due to methodological limits of the studies, particularly the lack of long-term follow-up to establish the durability and longevity of the observed beneficial effects and specific testing to establish whether the beneficial effects of tDCS observed in the laboratory/clinic generalized to everyday cognitive functioning and activities of daily living. Production of long-lasting and generalizable cognitive improvement by tDCS is essential to ensure clinical significance and meaningfulness of the benefits.

The field may benefit from drawing up some guidelines for application of tDCS as a therapeutic approach for NCDs.

## Author contributions

Study conception and design: DC, MJ, PB. Acquisition of data: DC. Analysis and interpretation of data: DC, PB. Drafting of manuscript: DC, PB, and MJ.

## Funding

PB is funded by the Bial Foundation (GA 84/2012).

### Conflict of interest statement

The authors declare that the research was conducted in the absence of any commercial or financial relationships that could be construed as a potential conflict of interest.
